# The Structure, Function, and Adaptation of Lower-Limb Aponeuroses: Implications for Myo-Aponeurotic Injury

**DOI:** 10.1186/s40798-024-00789-3

**Published:** 2024-12-24

**Authors:** Scott Hulm, Ryan G. Timmins, Jack T. Hickey, Nirav Maniar, Yi-Chung Lin, Katherine R. Knaus, Bryan C. Heiderscheit, Silvia S. Blemker, David A. Opar

**Affiliations:** 1 School of Behavioural and Health Sciences, Australian Catholic University, Melbourne, 115 Victoria Parade, Fitzroy, VIC 3065 Australia; 2 Sports Performance, Recovery, Injury and New Technologies (SPRINT) Research Centre, Australian Catholic University, Melbourne, 115 Victoria Parade, Fitzroy, VIC 3065 Australia; 3 Department of Sport Science and Nutrition, Maynooth University, County Kildare, Ireland; 4 Department of Bioengineering, University of California San Diego, La Jolla, CA USA; 5 Badger Athletic Performance Program, Department of Orthopedics and Rehabilitation, University of WI‑Madison, Madison, WI USA; 6 Department of Biomedical Engineering, University of Virginia, Charlottesville, VA USA

**Keywords:** Aponeurosis, Muscle–tendon injury, Morphology, Mechanical behaviour, Remodelling

## Abstract

The aponeurosis is a large fibrous connective tissue structure within and surrounding skeletal muscle and is a critical component of the muscle–tendon unit (MTU). Due to the lack of consensus on terminology and the heterogeneous nature of the aponeurosis between MTUs, there are several questions that remain unanswered. For example, the aponeurosis is often conflated with the free tendon rather than being considered an independent structure. This has subsequent implications when interpreting data regarding the structure, function, and adaptation of the aponeuroses from these studies. In recent years, a body of work has emerged to suggest that acute injury to the myo-aponeurotic complex may have an impact on return-to-sport timeframes and reinjury rates. Therefore, the purpose of this review is to provide a more detailed understanding of the morphology and mechanical behaviour common to all aponeuroses, as well as the unique characteristics of specific lower-limb aponeuroses that are commonly injured. This review provides the practitioner with a current understanding of the mechanical, material, and adaptive properties of lower limb aponeuroses and suggests directions for future research related to the myo-aponeurotic complex.

## Key points

1. In the current body of literature, there is inconsistency in the terminology and classification used to describe and study aponeuroses. This has subsequent implications when interpreting data regarding specific aponeurotic structures.
2. Injuries to the MTU also commonly involve damage or disruption to the aponeurosis and these injuries have often been associated with longer return-to-play times and have higher rates of re-injury compared to isolated muscle injuries
3. For clinicians it is important to understand the structure, function, and adaptive properties of aponeuroses as this may provide motivation for future research to direct myo-aponeurotic injury rehabilitation and prevention strategies.

## Background

Movement is a fundamental function of human existence. Skeletal muscles produce movement by generating contractile force, that is transmitted to adjacent bones, causing rotation around joints. This force is transmitted from contracting muscle to bone via a connective tissue network [1–3], that includes the free tendon and aponeurosis. Together, the muscle and its associated connective tissue are referred to as the muscle–tendon unit (MTU).The MTUs function is intimately related to its constitutive parts (i.e., skeletal muscle, tendon, and aponeurosis) and this intricate structure appears vital for performing dynamic tasks such as walking, running, and jumping [4–8]. The viscoelastic structure of the MTU plays an important role modulating the interrelationships between force output, muscle–tendon length changes and contraction velocity [9]. The structure of the MTU is plastic, in that it can be altered by mechanical stimuli in many ways, including training interventions [10–15], injury [16–20], immobilisation [21–24], and ageing [19, 22, 25–27].

Skeletal muscle is the most well researched component of the MTU [28–31]. However, the passive properties of muscle tissue are comparatively understudied relative to the active properties [32]. The relationship between skeletal muscle structure and function has been explored and described in detail [28–31]. Different skeletal muscles share common mechanical and physiological properties and demonstrate similar hierarchical organization from the microscopic to macroscopic level. However, the architecture (i.e., fascicle length, pennation and cross-sectional area (CSA)) [33], fibre typology (i.e., fast-twitch or slow-twitch) [34, 35] and neural control (i.e., motor unit number and size) [36, 37] are unique between muscles of different MTUs and are key determinants of force production. These differences also dictate how the contractile dynamics influence the role of the muscle unit, including the intrinsic force–length and force–velocity properties, that help optimise the control of human movement [38].

The free tendon is another integral part of the MTU that has been well researched in terms of its passive properties [14, 19, 26, 39]. is primarily without any inserting muscle fibres, made up of dense fibrous connective tissue predominantly composed of collagen, that facilitates force transmission from muscle to bone. Due to its viscoelastic properties, tendons can efficiently store and release energy during movement; contributing to the performance of motor tasks [6, 40]. As a result, tendons are exposed to high mechanical demands that lead to adaptation of the structural organization and the load-bearing capacity of the tendon tissue [13, 15, 41].

Compared to skeletal muscle and the free tendon, the aponeurosis is less well described within the literature, despite being a sizeable, fibrous connective tissue structure that links the muscle and free tendon. However, there is considerable heterogeneity within the research in regard to terminology – with the aponeurosis sometimes being described as the ‘central tendon’, ‘intramuscular tendon’, ‘rachis’, ‘septum’ or ‘inter-tendinous’ [42–45]. In many studies the aponeurosis is often conflated with the free tendon or is not considered as an independent structure. However, this assumption may not be valid as the tendon and aponeurosis are likely not mechanically in-series in a muscle fibre–aponeurosis–tendon model [46]. In fact, the aponeurosis may be best not bound by either a series or parallel designation due to its complex three dimensional behaviour [46, 47]. The aponeurotic structure is rather heterogeneous across MTUs which likely has implications regarding its mechanical role and its propensity for adaptation.

The aponeurosis plays a vital role in muscle–tendon function and is highly variable across MTUs in humans. Given the muscle-specific nature of the aponeurotic structure–function relationship, in this review, we concentrate on the human aponeuroses in the lower limb (excluding the foot), with specific emphasis on those acutely injured [45, 48–53]. This review aims to describe the common structural characteristics shared by human aponeuroses within the MTU and explores the range of different aponeurosis morphology. Additionally, the review delves into the current understanding of the mechanical, material, and adaptive properties of lower limb aponeuroses, while suggesting future research directions pertaining to the myo-aponeurotic complex. For this review we have primarily focused on human studies as aponeuroses are highly dependent on their structural and mechanical environment. Because lower limb MTU morphology differs greatly between humans and animal species in which aponeuroses have been studied [54, 55], human aponeurosis is likely to have a different mechanical function than the animal aponeurosis. From a clinical perspective, understanding this structure–function relationship is crucial, as injuries to human aponeuroses may require rehabilitation approaches tailored specifically to their unique biomechanical properties.

## Main Text

### Aponeurotic Injuries

Across many sports, hamstring injuries (particularly biceps femoris long head) are the most common muscle strain injury of the lower extremity and consequently, the most well-studied and characterized [56]. Following hamstring injuries, calf injuries (affecting the gastrocnemius and soleus muscles) and quadriceps injuries (predominantly rectus femoris) are notably prevalent, though they have received less research focus compared with hamstring injuries [57–59]. Injuries to these MTUs often involve some damage or disruption of the aponeurotic tissue; however, attention is largely devoted to muscle and tendon. As a result, aponeurotic injuries are often misconstrued in the literature. For example, many studies describe injury ‘at the muscle–tendon junction’ and fail to recognise that the free tendon coalesces with the muscle via the intricate aponeurotic and extra-cellular matrix (ECM) network [51, 60]. The aponeurotic tissue itself is commonly disrupted to varying severity [51, 61–63] and location along the length [49, 51, 61, 63, 64] of the myo-aponeurotic complex in association with acute muscle strain injuries [45, 60, 65–67]. A recent consensus statement highlights the importance of differentiating the type and location of myo-aponeurotic junction injuries, as this may have downstream effects influencing prognosis and risk of reinjury [61].

Myo-aponeurotic injuries can be conceptualised as occurring peripherally or centrally [61]. Peripheral injuries occur in an aponeurosis that covers the muscle surface, such as the distal aponeurosis of biceps femoris long head (BFlh) [52, 68], posterior aponeurosis of the rectus femoris [61] and the anterior aponeurosis of the gastrocnemius [51]. Central injuries occur in intramuscular aponeuroses that are surrounded on all surfaces by muscle fascicles and perimysium, but not epimysium [60]. Disruption can occur in aponeuroses that are either entirely intramuscular (such as the central aponeuroses of the soleus [50] or rectus femoris [49]), or only partially intramuscular (such as the intramuscular portion of the proximal aponeurosis of the BFlh [45, 66]).

Some myo-aponeurotic injuries are direct or isolated to the aponeurosis [61, 62]. The orientation of the disruption to the aponeurotic tissue can be described as transverse, longitudinal (split), or mixed [61]. The orientation of tissue disruption is significant; for example transverse injuries result in retraction of the muscle but isolated longitudinal injuries do not [61]. Further, it is speculated that longitudinal aponeurotic injuries are more likely to reinjure than transverse injuries due to the fact there is less fibrous repair [61]. In contrast, other work has found injuries with transverse and/or mixed aponeurotic disruption that leads to a scar tissue callus gap or transverse loss of continuity on MRI may also be associated with a higher risk of re-injury [69]. Other myo-aponeurotic injuries involve the junction between the aponeurosis and the muscle fibre and its perimysium [60, 61]. A small muscle fibre injury can cause focal disruption of the coalescing aponeurosis, while a major muscle injury can cause the muscle to shear away from its overlying aponeurosis, leaving a ‘gap’ between the intact aponeurosis and the injured muscle [51, 61].

In athletic populations, longer return-to-sport times after aponeurotic injury have been reported compared to isolated muscle injuries [48, 65, 70–72]. However, these return-to-sport times are typically shorter than those observed for the more burdensome, though less frequent, free tendon rupture or avulsion injuries [48, 73–78]. In a recent study of elite Australian Football players, acute hamstring injuries with high-grade (e.g. ‘3c’ per the British Athletics Muscle Injury Classification (BAMIC) system [62]) aponeurosis disruption were associated with significantly longer return-to-play times (88 days) compared to those with less severe aponeurotic injuries or no aponeurotic disruption (14–21 days) [70]. These findings are consistent with other studies of Australian Football players [65], elite track and field athletes [48, 71, 73] and professional soccer players [64, 72, 79]. Prolonged absences are not unique to aponeurotic injuries of the hamstring muscles. Triceps surae injuries with severe aponeurotic disruption have also been found to delay return to sport [43, 50, 51, 80]. Further, rectus femoris muscle injuries involving the central aponeurosis (including the intramuscular component, also known as a ‘de-gloving’ injury) have been shown to require longer periods of recovery [53, 74]. However, the potential bias in these studies should be noted as there was no mention of blinding to imaging findings (inclusive of magnetic resonance imaging or diagnostic ultrasound) for clinicians responsible for rehabilitation and return to sport decision making [43, 48, 50, 51, 53, 65, 70, 71, 81]. The only study to blind clinicians to imaging findings still demonstrated prolonged return-to-sport time when the aponeurosis was completely disrupted; however, no statistically significant difference was found between partial rupture and no disruption of the aponeurosis [79].

In addition to the reported longer return-to-play times, aponeurosis injuries may also be more likely to recur [43, 48, 64, 69, 70, 74]. A number of studies found the re-injury rates of elite track and field, football and soccer athletes were significantly higher for injuries with aponeurotic disruption, compared to those without aponeurotic involvement [43, 48, 69, 74]. The re-injury rate ranged from 31 to 63% in moderate aponeurotic injuries [48, 64, 70] and 33–57% in severe aponeurotic injuries [48, 64, 70], whilst re-injury rate of myo-aponeurotic junction injuries was 6–13%[48, 64, 70]. However, two other studies have described alternative findings, with no significant increase in re-injury rate within 12 months of return to sport [44, 82]. The discrepancy may be due to differences in aponeurosis injury classification BAMIC [62] vs a standardised scoring method of aponeurosis disruption [44, 79]), type of athlete (e.g. elite sprinters [48, 74] vs footballers [44, 82]),the time period observed (e.g. 1-year [79] vs. 4-year re-injury observation [48]) or the injury rehabilitation approach.

Given that the aponeurosis is a common site of injury, with studies showing extended rehabilitation times and high re-injury rates, a detailed understanding of its structure, mechanical behavior, and capacity for adaptation is essential. This knowledge is crucial for addressing these types of injuries and guiding future research on rehabilitation and prevention.We acknowledge the existence of additional research in animal [83, 83, 84] and in-silico models [3, 85, 86] regarding alterations in morphology and mechanical behaviour following aponeurotomy (induced aponeurotic disruption). While our review focuses on human data and does not delve into these specific aspects, we recommend the following studies to interested readers for further insight following aponeurotic disruption [3, 83–88].

### Literature Search Strategy

A retrospective, citation-based methodology was implemented, per previous literature [89, 90] to discover English-language literature relating to (1) the structure of human lower-limb aponeuroses; (2) the mechanical behaviour of human lower-limb aponeuroses; (3) the morphological, mechanical, or material adaptations of human lower-limb aponeuroses following intervention; and (4) aponeurotic injury. The articles included in this review were obtained from PubMed from database inception to August 2022. Due to interchangeable terminology on this topic, the search strategy was intentionally kept broad to avoid omission of relevant studies. Studies that focused on the aponeuroses of the upper limb, trunk and foot, or studies that did not delineate the aponeurosis from its attached free tendon in their methods were not included. Where appropriate, weighted means (as described in Tables 1 and 2) were used to aggregate the data where there was sufficient homogeneity across the variable of interest. One author (SH) independently screened and documented the literature (see supplementary Appendix S1 for search keywords).

**Table 1:** Aponeurotic dimensions for the triceps surae muscles

Aponeurosis		Length (mm)	Width (mm)	CSA (mm^2^)	Thickness (mm)	Type
Anterior	Medial Gastrocnemius	9.2 ± 11.8			0.52 ± 0.02^a^	Cadaver [118, 119, 121]
	Lateral gastrocnemius	26.7 ± 22.4			0.42 ± 0.07^a^	Cadaver [118, 119, 121]
	Medial Soleus	187.0 ± 24.2	74.2 ^b^		0.45 ± 0.01^a^	Cadaver [92, 118, 121] MRI [47, 50]
	Lateral Soleus	171.5 ± 11.6	74.2 ^b^		0.51 ± 0.01 ^a^	Cadaver [92, 118, 121] MRI [47, 50]
Posterior	Medial Gastrocnemius	187.5 ± 33.1		38.7 ± 7.2	0.47 ± 0.03^a^	Cadaver [118, 120, 121] US [122, 123]
	Lateral gastrocnemius	194.0 ± 11.3		16.7 ± 4.0	0.48 ± 0.01^a^	Cadaver [118, 120, 121] US [122]
	Medial Soleus	230.0 ± 34.4	88.1 ^b^		0.35 ± 0.03^a^	Cadaver [118, 121] US [122] MRI [47]
	Lateral Soleus	230.0 ± 34.4	88.1 ^b^		0.40 ± 0.04^a^	Cadaver [118, 121] US [122] MRI [47]
	Central Aponeurosis (Median Septum)	213.1 ± 68.2	31.8 ± 0.5			Cadaver [92] MRI [47, 50]

For those dimensions with multiple literature sources, we calculated a weighted mean and standard deviation which considered sample sizes across studies. If more than one measurement was reported in a literature source, the numbers were averaged. If more than one measurement location was available for the literature source (e.g., proximal, middle, distal), the numbers were averaged. Aponeurosis width measurements list was defined as the curvilinear distance in the medio-lateral direction [47, 118]. The average thickness of each aponeurosis listed were calculated after measuring the thickness at four different sites by using a digital vernier caliper in the anterior to posterior direction [118]. The average thickness and width of the cadaveric aponeurosis specimens were used to calculate the cross-sectional area of the measurements listed below [120]

^a^Dimensions approximated from digitisation of graphical data. CSA, Cross sectional area; MRI, magnetic resonance imaging; US, ultrasound. ^b^The source did not differentiate between the medial and lateral aponeuroses values reported

**Table 2:** Aponeurotic dimensions for the hamstring muscles

Aponeurosis		Length (cm)	Width (cm)	Thickness (cm)	Interface area (cm^2^)	Volume (cm^3^)	Type
BFlh	Proximal	18.3 ± 3.0	0.9 ± 0.3	0.2 ± 0.07	24.4 ± 11.2	2.9 ± 0.7	Cadaver [124, 125, 129, 130] US [93, 129] MRI [115–117, 125, 127, 128, 131]
	Distal	17.9 ± 1.6	4.6 ± 0.3	0.2 ± 0.04			
BFsh	Distal	10.7 ^a^					Cadaver [124]
ST	Proximal	11.8 ± 0.7			18.8 ± 3.7		Cadaver [124, 125, 130] MRI [125]
	Aponeurotic inscription	8.5 ± 1.1					
	Distal	12.0 ± 0.8					
SM	Proximal	18.3 ± 3.2			84.6 ± 31.5		Cadaver [124, 125] MRI [125]
	Distal	17.0 ± 1.1					

For those dimensions with multiple literature sources, we calculated a weighted mean and standard deviation which considered sample sizes across studies. If more than one measurement was reported in a literature source, the numbers were averaged. If more than one measurement location was available for the literature source (e.g., proximal, middle, distal), the numbers were averaged. Aponeurosis width measurements were defined as the curvilinear distance in the circumferential direction with respect to the center of the muscle’s anatomical cross section [115]. Aponeurosis thickness measurements were defined as the area divided by the width measurement [115]. Interface area was defined as the contact interface distance between the muscle and the proximal aponeurosis outlined in each image/specimen where the aponeurosis was identifiable and multiplied by the slice thickness [125, 127, 128]

^a^No standard deviation reported. MRI = magnetic resonance imaging. US = ultrasound. BFsh = biceps femoris short head. ST = semitendinosus. SM = semimembranosus.

## Structure

Aponeuroses have a complex three-dimensional geometry that differs significantly across MTUs and serve as an intricate insertion site for muscle fascicles, influencing their orientation and pennation angle [91–94]. As such, variation in the geometrical structure of aponeuroses between individuals, between different MTUs and following adaptation will influence the mechanical behaviour of the aponeurosis and may provide some rationale for injury at these sites. The following sections will outline the structure and variation of aponeurotic structures.

### Aponeurosis Macrostructure

**Fig. 1:**
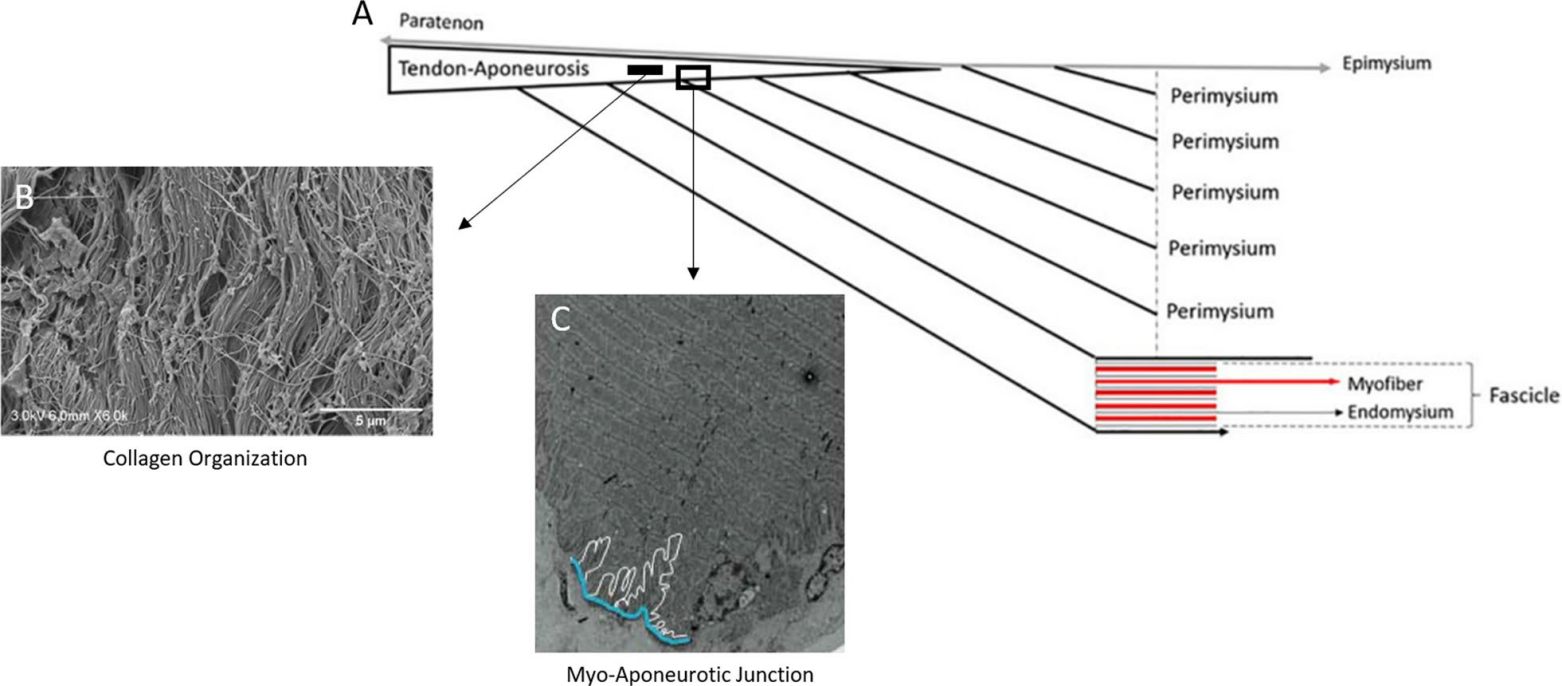
**A** A connective tissue continuum from the tendon-aponeurosis via the union of the perimysium and epimysium to the aponeurosis. The endomysium (grey line and dark grey arrow) surrounds each individual myofiber (red line and arrow), the perimysium surrounds several fascicles of myofibers (vertical grey dashes) and the epimysium surrounds the entire muscle (light grey line and arrow) [60]. Reproduced and adapted from Balius et al.[61] with permission from SAGE publications. **B** The collagen organization of porcine aponeurotic tissue involves branching of obliquely and transversely orientated collagen fibres in conjunction with the longitudinally arranged collagen sheet [101]. Reproduced and adapted from Grega et al.[101] with permission from Elsevier. **C** The surface area and base length of the myo-aponeurotic junction. The ridge-like protrusions are outlined in white with the base length of the MTJ outlined in blue [102]. Reproduced and adapted from Knudsen et al.[102] with permission from John Wiley and Sons

The aponeurosis often appears as a sheet-like extra and/or intramuscular section of connective tissue, that serves as an insertion interface for muscle fibres. Aponeuroses are continuous with the free tendon and directly link with muscle tissue and the extra-cellular matrix (ECM) (e.g., epimysium and perimysium), forming a continuous myo-aponeurotic unit (Fig. 1A) [31, 60, 84, 95]. The ECM is a complex organization of fibrous collagen networks, distinctly divided into three layers: endomysium, perimysium, and epimysium [31, 61]. Within this architecture, the endomysium envelops each individual myofiber and primarily consists of collagen types I, III, and V [96]. The perimysium encases several fascicles of myofibers and is predominantly composed of collagen types I and III [96]. On a larger scale, the epimysium surrounds the entire muscle, with its composition primarily consisting of collagen types I and III [96]. The extramuscular aponeuroses are extensions of the free tendon that appear superficially to the muscle tissue before coalescing with the epimysium, while the intramuscular aponeuroses are surrounded on all surfaces by muscle fibres and perimysium, but not epimysium [51, 61].

The interface between the aponeurosis, contractile elements, and ECM is pivotal for force transmission within the MTU. Healthy terminating muscle fibers primarily undergo shear forces through a rigid endomysium, while the shear-compliant perimysium allows fascicles to slide past one another during muscle contraction [97, 98]. Previous mathematical models have demonstrated that terminating muscle fibers primarily transmit force through shearing of the endomysium rather than tension [98]. These shear forces distribute force across the fiber surface, while tensile forces only act on a smaller endomysium area [98]. A thin endomysium results in significant shear strains and substantial shear forces opposing fiber shortening [98]. Other computational models of fascicle shape and size revealed that the thickness of the perimysium and fascicle arrangement influence muscle properties [97]. These simulations indicated that the perimysium experiences higher shear strains than fascicles, reinforcing the notion that the perimysium adjusts to accommodate the diverse shear strains required for muscle function [97]. Additionally, significant shear strains between fascicles in human pennate muscles have been described, which suggests significant variation between different MTUs [99].

Furthermore, finite element models of the myo-aponeurotic junction also investigated the local microscale strains in terminating muscle fibers [100]. These models found that microscale strains increase with muscle activation and endomysium stiffness. The endomysium appears to act as a passive resistance during active muscle lengthening, balancing forces and preventing excessive strain at the muscle fiber termination at the aponeurosis interface [100]. This suggests that the endomysium may contribute to safeguarding muscle from injury by reducing muscle fiber strains at the myo-aponeurotic junction.

### Aponeurosis Microstructure

Healthy aponeurotic tissue exhibits a striated appearance comprised of longitudinally-arranged fibroblasts and type I collagen fibres [50, 84] similar to that of tendons and ligaments. Some aponeurotic collagen fibres may also be obliquely or transversely oriented [103–105] with a unique ‘crimped’ and ‘wavy’ pattern (Fig. 1B) [84, 95, 101]. This orientation of collagen fibres may straighten or ‘uncrimp’ during aponeurosis elongation both longitudinally and transversely, which may influence the aponeurosis stress–strain relationship biaxially [101]. At the ECM interface the collagen fibres have a more reticular appearance, being fine and randomly oriented, possibly because the aponeurosis accumulates muscle forces from different angles of fascicle pennation [42, 106].

### Myo-Aponeurotic Junction Morphology

Although the term ‘muscle–tendon junction’ (MTJ) is widely used throughout the literature, it is important to acknowledge that there are alternative perspectives regarding the precise definition and structural characteristics of this interface [60–62, 107, 108]. For many MTUs, the MTJ might be more accurately described as the myo-aponeurotic junction, as it is often the interface between the muscle fibres and its aponeuroses.The myo-aponeurotic junction appears as a series of ridge-like connective-tissue foldings that protrude into the muscle tissue. These foldings penetrate the muscle membrane and are directed towards the myofibrils (Fig. 1C) [107, 109]. This morphology serves to increase the contact interface between a muscle and its aponeuroses, thereby distributing force over a greater surface area [109]. This constituent of the MTU is important as it is exposed to the highest force transmission [100, 110] and yet has been found to be mechanically the weakest part of the MTU in some cases [111, 112]. This may be the reason for the large number of strain injuries involving the myo-aponeurotic junction [45, 113].

### Morphological Variation of Aponeuroses

#### Between Muscle Variation

Lower-limb aponeuroses are variable between muscles due to the variability in muscle architecture and therefore the different functional requirements of individual MTUs. The aponeuroses of the triceps surae and hamstrings, for example, have unique features that serve as useful case examples of the distinct anatomic variability between muscle groups (Table 1, 2). These specific aponeurotic arrangements are likely to have implications for function, adaptation, injury or remodelling of specific myo-aponeurotic interfaces [32, 47, 114–117].

**Fig. 2:**
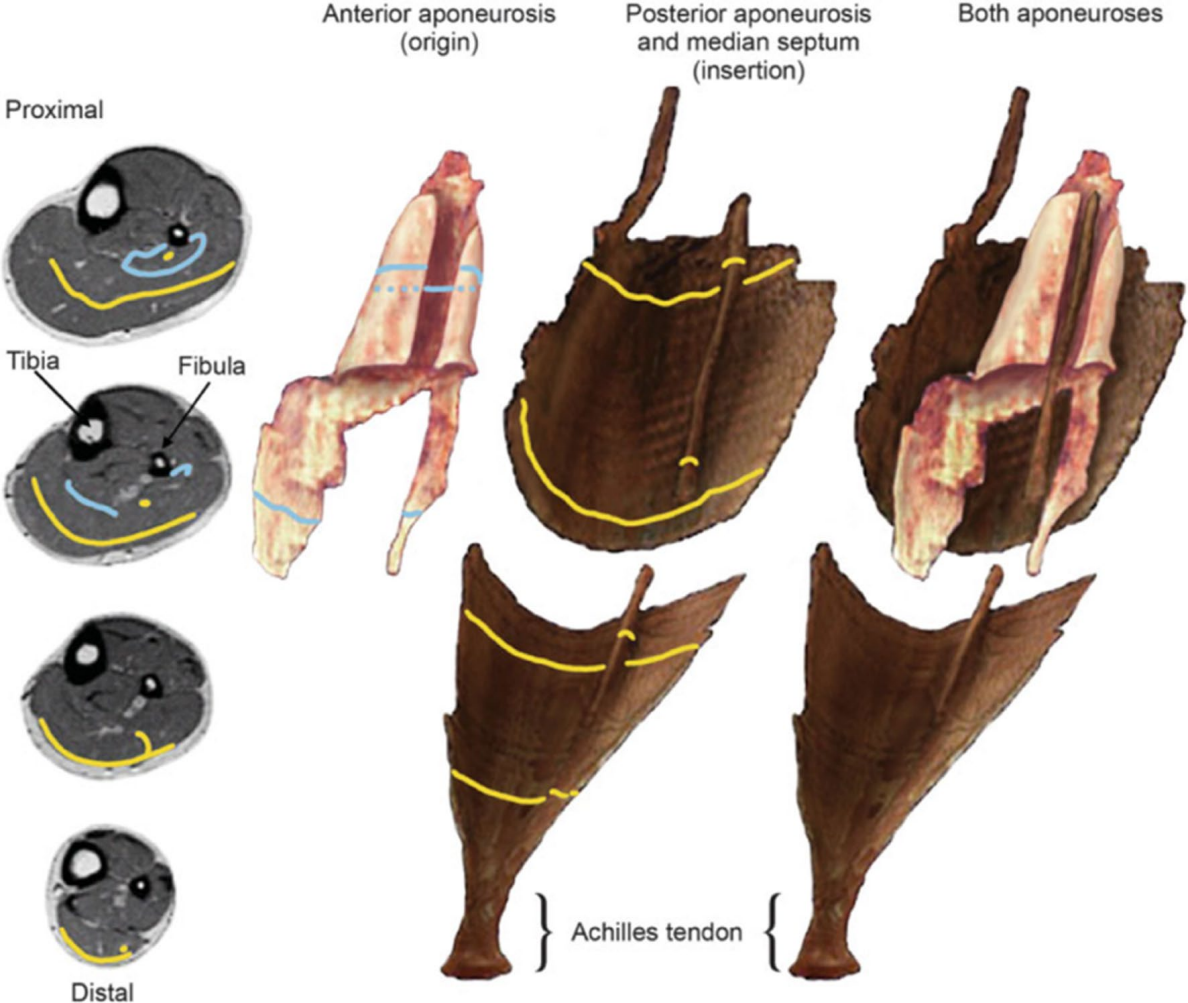
Three-dimensional model images of the soleus aponeurosis from MRI images (viewed anteriorly). The blue and yellow lines in the axial images (left) correspond to those in the 3D structures (right). The images on the right demonstrate the attachment of the central aponeurosis to the posterior aponeurosis before projecting anteriorly to interdigitate with the anterior compartment. Reproduced from Hodgson et al.[91] with permission from John Wiley and Sons

The three aponeuroses of the soleus (anterior, posterior and central aponeuroses) are unique in that they are interdigitating (i.e., interlocking like the fingers of two clasped hands). The anterior aponeurosis forms a deep trough at the region where the central aponeurosis separates from the posterior aponeurosis. At this site the central aponeurosis penetrates the anterior aponeurosis to form the insertion site for the muscle fibers of the anterior compartment (Fig. 2) [91]. Injuries involving the central aponeurosis of the soleus muscle have been shown to require longer recovery and return-to-play times compared to those that spare the central aponeurosis which may be a reflection of the complex anatomy at this site.[80].

**Fig. 3:**
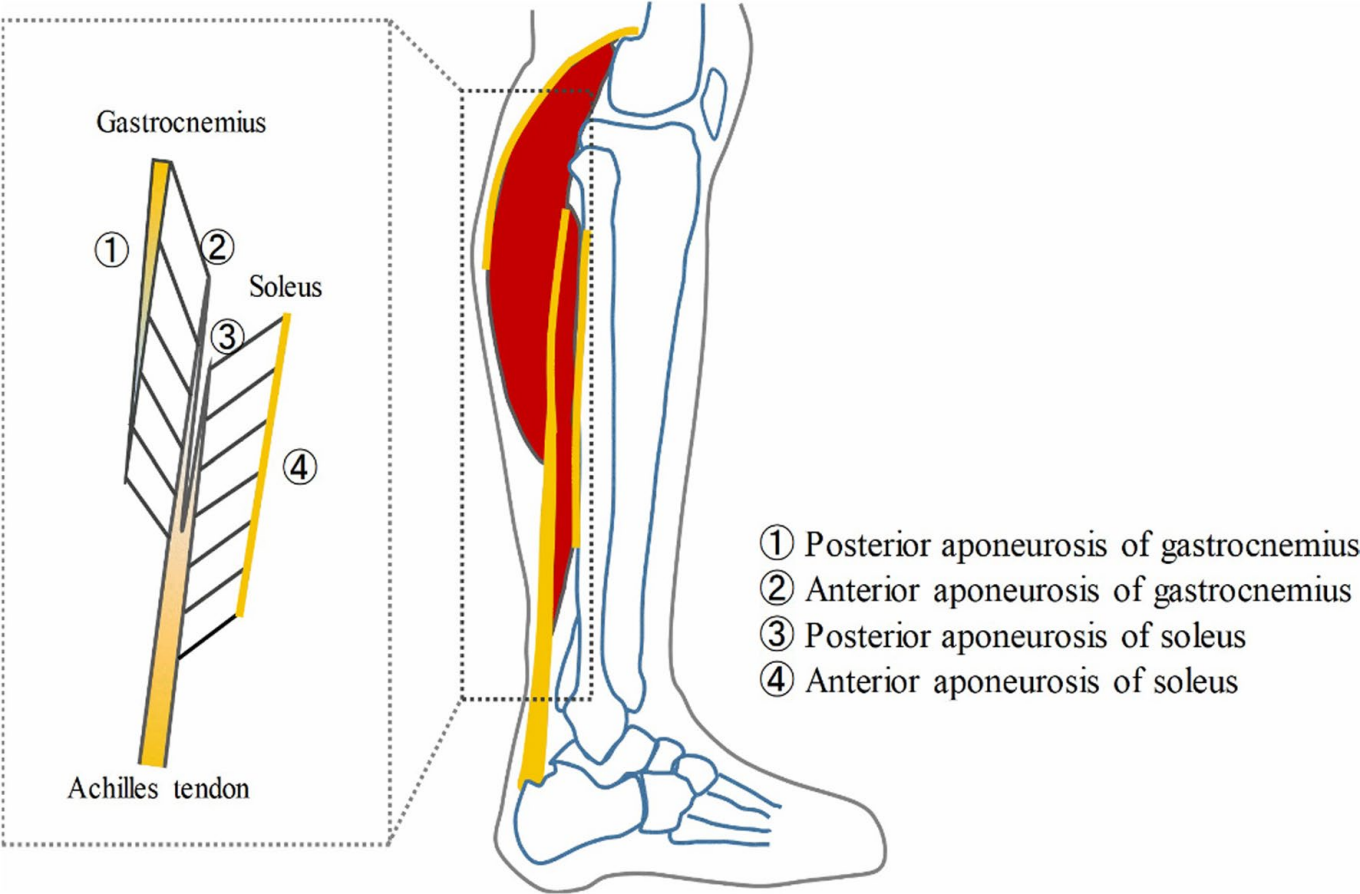
Schematic diagram of the triceps surae aponeurotic structure from the sagittal view [118]. Reproduced from Shan et al.[118]. This work is licensed under a Creative Commons Attribution 4.0 International License

The gastrocnemius muscle, in contrast, is comprised of two aponeuroses (anterior and posterior) [118] which do not interdigitate. The anterior aponeurosis of the gastrocnemius muscle arises from the Achilles tendon and courses superiorly. On imaging, it appears to be in direct contact with the posterior aponeurosis of the soleus, however; they are in fact completely separate over most of their lengths (Fig. 3) [118, 119]. Injuries along the medial gastrocnemius anterior aponeurosis are relatively common and have been linked to longer return-to-sport times in athletes, especially when there is a more severe lesion at the myo-aponeurotic junction or within the aponeurosis itself [51].

**Fig. 4:**
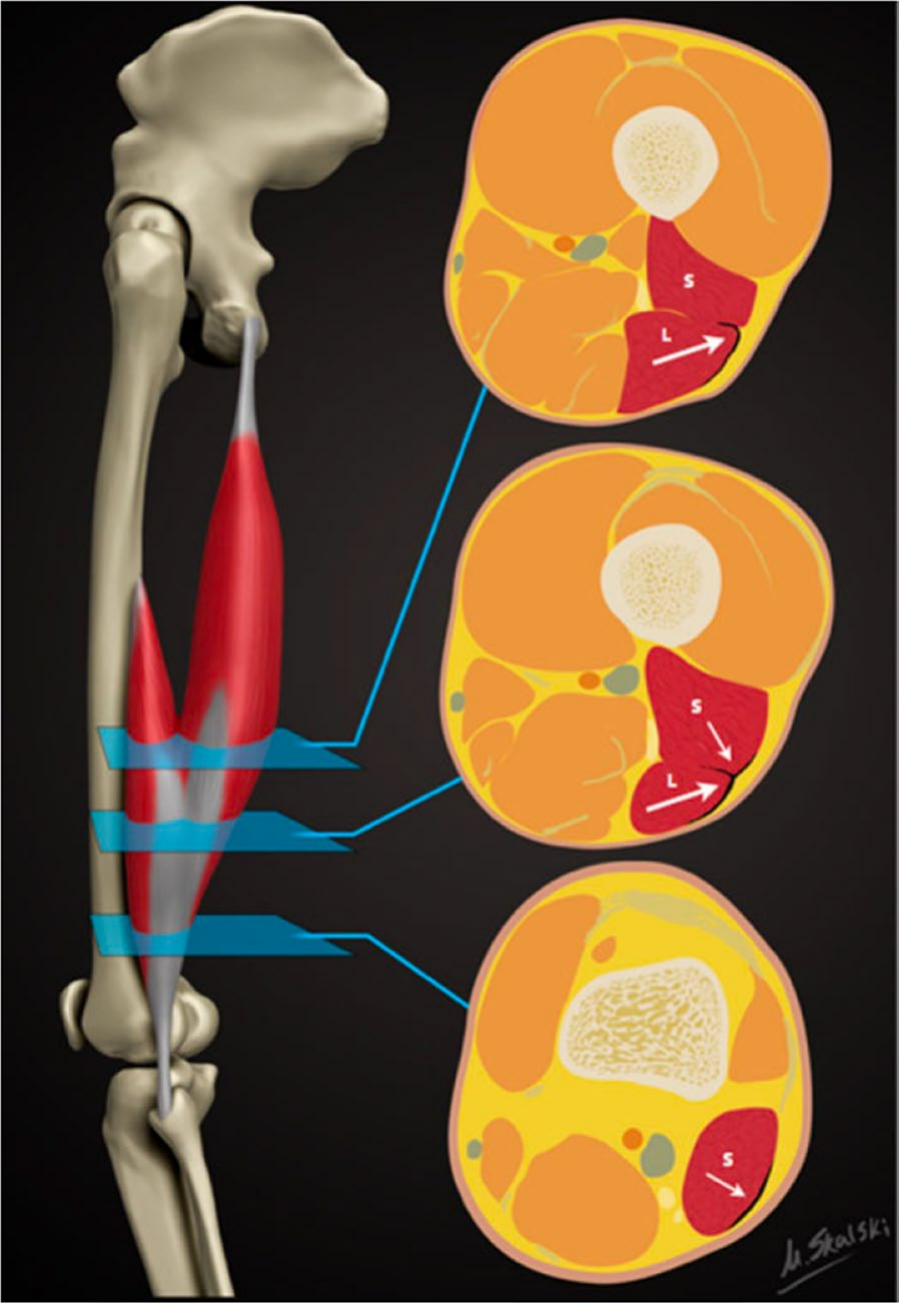
Illustration demonstrating the regional axial variations of the distal aponeurosis of the biceps femoris [52]. Proximally, the large white arrow illustrates the myo-aponeurotic interface at the distal aponeurosis with biceps femoris long head (L) and may appear L-, C-, or U-shaped. In the middle slice, the distal aponeurosis appears as a T-shaped structure and acts as the myo-aponeurotic interface for both biceps femoris long head (L, large white arrow) and short head (S, small white arrow). Distally, the distal aponeurosis appears as a shallow convex structure and the small white arrow illustrates the myo-aponeurotic interface with biceps femoris short head (S). Reproduced from Entwisle et al.[52]. This work is licensed under a Creative Commons Attribution 4.0 International License

The aponeuroses of the hamstrings muscles also demonstrate a unique anatomy. Each of the hamstring muscles (BFlh, biceps femoris short head (BFsh), semimembranosus) are comprised of two aponeuroses (proximal and distal), except for the semitendinosus muscle which has a distinctive third aponeurotic structure otherwise known as the ‘inscription’. This is a projection in the shape of an inverted ‘V’ which divides the muscle into superior and inferior regions [124]. Another unique feature of the hamstrings is that the proximal aponeuroses of the BFlh and the semitendinosus are closely interrelated, forming a conjoined or shared aponeuroses [124–126]. The proximal aponeurosis of the BFlh is further distinctive in that it then courses inferiorly within the muscle belly and becomes intramuscular [124–126]. Importantly, the proximal myo-aponeurotic junction and the proximal aponeurosis itself are frequent sites of disruption along their length in hamstring strain injuries across professional sports [48, 56, 64, 70, 73]. Finally, the distal aponeuroses of both the BFlh and BFsh merge to form a T-shaped structure in the mid-section of the muscles (Fig. 4) [52, 64, 68]. It appears this complex anatomic structure of the distal aponeurosis has hamstring injury consequences, as injuries involving this particular aponeurotic structure have been described as highly recurrent with a ~ 50% reinjury rate reported across studies [52, 64, 68].

**Fig. 5:**
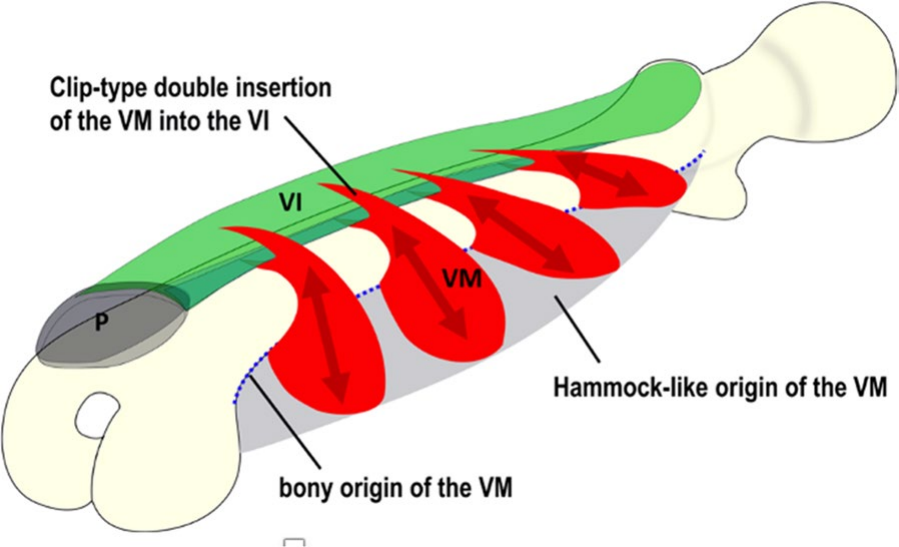
Schematic drawing of the clip-type connection between vastus medialis (VM) and vastus intermedius (VI). The hammock-like aponeurotic origin (grey) and clip-type double insertion (red) of VM into the VI aponeurosis (anterior and posterior aspects) (green) are shown [132]. Reproduced from Grob et al.[132] with permission from Springer Nature

While the aponeuroses of the triceps surae and hamstrings muscles are the most widely studied, other muscle groups also display distinctive characteristics. For example the vastus medialis originates from its medial aponeurosis then traverses laterally to merge with the vastus intermedius aponeurosis in what has been described as a ‘clip-type’ insertion, as a paperclip would attach to a sheet of paper (Fig. 5) [132]. The rectus femoris, as another example, has a complex structure, owing to its two long aponeuroses (anterior and posterior) and an intricate intra-muscular central aponeurosis which gives rise to a unique ‘muscle within a muscle’ arrangement [49, 53, 133]. The central aponeurosis of rectus femoris is a common site of injury [49, 74, 133] and the severity of aponeurotic disruption at this site has been associated with longer return to play times [74].

#### Regional Variation Within Aponeuroses

Aponeuroses often display a non-uniform geometry along their length. For example, in many of the MTUs described in the previous section, the aponeurosis has different dimensions at specific transitional regions such as the aponeurosis-bone, the aponeurosis–tendon, and the myo-aponeurotic junctions. This regional variation accommodates different stresses and strains at each site and may serve to prevent or increase the likelihood of damage [115, 118, 121].

Among many lower-limb muscles, the proximal and distal aponeuroses generally tend to narrow and thicken at the junction with the free tendon, and widen and flatten as they coalesce with muscle tissue in the muscle belly [115, 116, 124, 125]. Further, the widths of the BFlh aponeuroses vary along their lengths [115, 116] (as do presumably other aponeuroses of the lower limb)(Table 2) For example, the proximal aponeurosis of BFlh is still relatively much narrower at the point at that it coalesces with the muscle, than the broad, sheet-like distal aponeurosis [115, 124]. A smaller ratio between the proximal aponeurosis width and muscle widths has been estimated to increase the magnitude of local fibre strain within the BFlh during model simulations of high-speed running (see Fig. 6 in Fiorentino et al.[115]) [115–117]. This demonstrates that myo-aponeurotic regional variability has the potential to influence the internal tissue strain response during sprinting.

In general, aponeuroses are thicker where they arise from the free tendon and then taper as they merge with muscle tissue [118]. This is true for the proximal aponeurosis of the BFlh, the posterior aponeurosis of the soleus, as well as both the anterior and posterior aponeuroses of the gastrocnemius [115, 118, 121]. In contrast, the distal aponeurosis of the BFlh (thinner at its distal end where it coalesces with the free tendon [115]) and the anterior aponeurosis of the soleus (uniform thickness along its entire length [118]) do not follow this pattern. Regional variability has been linked with differences in the longitudinal stiffness properties of the gastrocnemius aponeuroses measured in cadavers [118]. These regional differences in thickness may potentially have a downstream effect on the site-specific strain response. Previous model simulations of BFlh have found that thicker and thinner aponeurotic tissue resulted in larger and smaller muscle fibre strains along the length of the myo-aponeurotic junction, respectively [117]. This may have implications for the regional strain response and strain injury location during active lengthening tasks such as sprinting.

#### Between Individual Variation

The morphology and geometry of each aponeurosis vary between individuals [50, 92, 114–116, 118, 119, 124, 125, 134]. This has a direct effect on muscle pennation angle, fascicle length and surface area for muscle fibre attachment [50, 92, 114, 119, 134]. For example, four distinct morphologies of the soleus and its associated aponeuroses have been described [92, 114]: a bipennate muscle with fascicles attached to both sides of a central aponeurosis; a unipennate muscle with fascicles attaching at an acute angle from one side of the central aponeurosis; a multipennate muscle in which fascicles attach to multiple intramuscular aponeuroses; and a non-pennate muscle without a central aponeurosis but with a wide aponeurotic surface. The gastrocnemius aponeuroses also demonstrate inter-subject variability. For example, the anterior aponeurosis has been described as ‘long’ (> 10 cm), ‘short’ (< 10 cm) or absent, where either one or both heads of the gastrocnemius insert directly into the posterior soleus aponeurosis [119].

Variability between individuals has also been demonstrated in the hamstring aponeuroses. Previous work demonstrated a coefficient of variation (CV) of 18% across all BFlh dimensions (including muscle and free tendon dimensions) and measurement locations (superior to inferior) [115]. For the BFlh proximal aponeurosis alone, the CV was 18% for width and thickness measures [115] and 27% for cross sectional area [127]. This variability potentially has injury implications with more narrow BFlh aponeuroses being subject to greater strain at the proximal myo-aponeurotic junction than wider aponeuroses [116].

### Future Directions

The structure of the human aponeuroses demonstrates notable regional and individual specific variability along with muscle specific intricacies such as interdigitating components [91, 118, 119, 132] and shared aponeuroses [52, 68, 119, 134]. As such, future research into structure–function relationships of aponeuroses should be muscle specific and avoid conflation with other MTUs. Further investigation into the collagen structure of human aponeuroses – as has been done in animals via scanning electron microscopy [101] and image-based modelling [135]—is also necessary to understand its mechanical behaviour and potential for adaptation, particularly if collagen structure varies between aponeuroses of muscles with different functional behaviour.

Various methodological approaches have been employed by researchers to quantify the "size" of an aponeurosis in MR imaging, with no universally accepted reporting standard currently in place. The literature presents a range of measurement techniques, including the assessment of aponeurosis CSA [120], width [115, 116, 128, 136], thickness [117, 118], and volume [128], as well as metrics related to the contact interface between the aponeurosis and its associated muscle [125, 127, 128, 137, 138]. For instance, the width of the aponeurosis is often measured at a single location along its length [115, 116]. The thickness of the aponeurosis has been reported as the aponeurotic area divided by the width measurement due to the variability in thickness across its width [115]. In other studies, the aponeurosis interface area is calculated by multiplying the measured contact interface by the number of slices or by the aponeurosis length [125, 127, 128, 137, 138]. These varied methodologies yield fundamentally different evaluations of aponeurosis "size", potentially complicating the comparison of results across different studies.

The inter-connection between the ECM and aponeurotic structures also raises several unanswered questions regarding its structure. For example, it is unclear how the histoarchitecture differs between the epimysium and perimysium at the region they coalesce with the aponeuroses, particularly when considering multipennate muscles (e.g. soleus) with varying muscle to aponeurosis attachment angles [92] or those muscles with distinct intramuscular sections of their aponeurosis (e.g., distal intramuscular portion of the BFlh proximal aponeurosis [61]).

Inconsistencies in terminology used to describe aponeuroses and the myo-aponeurotic unit contribute to the lack of consensus on this topic and possibly add to the variability in findings pertaining to aponeurosis function, adaptation and injury. Future work should aim to define a histoarchitectural nomenclature of connective tissue structures that helps define anatomical structures of the aponeurosis in isolation – as has been done recently regarding myo-aponeurotic injuries [61]: for instance, a terminology that clearly defines the location of the aponeurosis relative to the muscle (e.g. extra-muscular or intra-muscular aponeuroses [51, 61]) and relative to its anatomical location (e.g. distal versus proximal aponeuroses). This will help to improve the consistency when assessing and reporting the mechanical behaviour of the specific aponeurotic tissue independently of the free tendon.

## Function

This section will focus on key mechanical and material properties from studies that have measured the aponeurosis independently of the free tendon, and excludes those that do not delineate the aponeurosis from its attached free tendon in their methods [139–141]. The presumption of this exclusion criterion is that the measured properties more accurately represent those of the aponeurosis [142, 143] compared to those that combine the tendon and aponeurosis [140, 141, 144].

### Biaxial Behaviour

A unique behaviour of the aponeurosis is its biaxial loading pattern during muscle contraction. This contrasts with the free tendon which primarily deforms uniaxially in a longitudinal direction [145]. The aponeurosis is exposed to biaxial loading due to the direct insertion of muscle fascicles onto aponeurotic tissue. As muscle fascicles contract, they must simultaneously expand radially to maintain a constant volume during force production [146]. Therefore, the aponeurosis undergoes longitudinal strain in the direction of tendon loading and transverse strain perpendicular to the muscle’s line of action [1] during active muscle contraction [147]. Under passive stretching; however, the aponeurosis primarily deforms uniaxially in the longitudinal direction [94, 148, 149], similar to the free tendon.

The mechanical and material properties in both the transverse and longitudinal directions appear to differ from the free tendon [94, 101, 118]. The ratio of transverse to longitudinal strain is significantly higher for the aponeurosis, compared to the free tendon [147, 150–152]. This may be due to variations in morphology [150–152], loading pattern (uniaxial vs biaxial) [153] or differences in the material properties biaxially [94, 101, 118].

Shan et al. [118] determined the elastic modulus (or Young’s modulus defined as the slope of the elastic region of the stress–strain curve) of the human triceps surae aponeuroses to be 0.5–3 MPa (at 5–30% strain) in the transverse direction, which was approximately 100 times less than that in the longitudinal direction (100–500 MPa, at 3–5% strain). These findings support that the aponeurosis is stiffer in the longitudinal direction compared to the transverse direction [94, 154] and that the aponeurosis is more compliant in the transverse direction in order to undergo greater transverse stretch necessary to accommodate the bulging muscle tissue during contraction [146]. However, further material testing of aponeuroses from different MTUs is needed to confirm this behaviour.

Patterns of biaxial deformation are potentially influenced by complex aponeurosis structure [150–152] such as the presence of obliquely or transversely oriented collagen fibrils within aponeurotic tissue [103–105]. The number of transversely oriented fibrils is modest compared to the dominant longitudinal structure, thereby potentially explaining the marked differences in mechanical and material properties in a transverse, compared to a longitudinal, direction [101, 118]. As the aponeurosis is loaded, the regions with wavy morphology may straighten or ‘uncrimp’ biaxially. This has been described as the mechanism for the non-linear or ‘toe-region’ in the longitudinal and transverse stress–strain relationships of connective tissues [101, 155, 156].

### Mechanical and Material Properties of Human Aponeuroses

The mechanical behaviour of aponeurotic tissue, including properties like strain and stiffness, depends not only on the level of muscle force but also on their morphological and material characteristics [46].Mechanical stiffness of the aponeurosis is a function of deformation and its material properties [46]. Stiffness has been reported previously as a change in aponeurosis dimensions (biaxial deformation) in relation to the force applied to the MTU in vivo; however as the aponeurosis force is not directly measured, these methods represent only an apparent aponeurosis stiffness [157]. In contrast, ex vivo studies measure the deformation of the aponeurosis in response to force being applied directly to the aponeurosis [118]. However, this does not accurately reflect in vivo aponeurosis behaviour as it does not represent the deformation that results from the interconnection between the contractile elements and passive tissue [46]. In this section we describe apparent stiffness where in vivo techniques have been used whereas for ex vivo we use the term mechanical stiffness and acknowledge the limitation of this measurement type. Both the mechanical properties (strain) and the material properties (modulus) will vary between different aponeuroses of separate muscle groups, within different regions of the same aponeurosis as well as between longitudinal and transverse loading regimes (refer to Tables 3, 4 and 5).

**Table 3:** Biaxial strain values of human lower-limb aponeuroses

Muscle group	Aponeurosis	Measure	Contraction mode	Velocity	MTU Length	Contraction intensity (MVIC unless otherwise stated)	Longitudinal strain (%)	Transverse Strain (%)	Reference
Medial Gastrocnemius	Anterior	US	Isometric			0%	0.05 ^a^		[158]
						15%	0.72 ^a^		
						35%	1.76 ^a^		
						50%	2.63 ^a^		
						60%	3.51 ^a^		
						80%	4.50 ^a^		
						100%	5.12 ± 2.07		
	Anterior	MRI	Isometric			20%	Proximal: 5.65 ^a^ Central: 2.54 ^a^ Distal: 4.99 ^a^		[159]
						40%	Proximal: 7.55 ^a^ Central: − 2.58 ^a^ Distal: 9.43 ^a^		
	Anterior	US	Isometric			100%	Proximal: 4.4 ± 0.5 Distal: 5.6 ± 0.4		[160]
	Anterior	US	Isometric			100%	1.4 ± 0.4		[161]
	Anterior	US	Passive	5⁰∙s^−1^	Short		− 0.5 ^a^		[162]
					Moderate		2.4 ^a^		
					Long		4.3 ^a^		
	Anterior	US	Passive	5⁰∙s^−1^	Short		− 1.8 ± 1.1		[163]
					Long		2.1 ± 1.1		
	Anterior^b^	MRI	Isometric			30%	1.1 ± 1.2	Proximal: 8.0 ± 9.4 Distal: 11.4 ± 1.1	[147]
						60%	1.6 ± 1.1	Proximal: 8.5 ± 10.6 Distal: 13.9 ± 11.0	
	Anterior^b^	US	Isometric			10%	2.97 ^a^		[164]
						20%	3.22 ^a^		
						30%	3.96 ^a^		
						40%	5.11 ^a^		
						50%	5.38 ^a^		
						60%	6.27 ^a^		
						70%	5.68 ^a^		
						80%	6.91 ^a^		
						90%	7.34 ^a^		
	Anterior^b^	US (3D)	Isometric			50%	3.0 ± 0.1		[165]
	Posterior	US	Isometric			100%	Overall: 5.6 ± 1.2 Proximal: 5.5 ± 1.3 Distal: 5.8 ± 1.8		[123]
	Posterior	MRI	Isometric			20%	Proximal: 5.10 ^a^ Central: 1.16 ^a^ Distal: − 2.14 ^a^		[159]
						40%	Proximal: 6.95 ^a^ Central:− 2.90 ^a^ Distal: − 10.26 ^a^		
Soleus	Posterior	MRI	Isometric			20%	Central: 2.0 ± 0.4^a^ Distal: 2.1 ± 0.7 ^a^		[166]
						40%	Central: 2.2 ± 1.7 ^a^ Distal: 2.5 ± 1.6 ^a^		
Tibialis anterior	Central	US	Isometric			100%	Overall: 6.5 ± 0.6 Proximal: 9.2 ± 1.0 Distal: 3.5 ± 0.3		[167]
	Central	US	Isometric			20%	2.1 ± 0.4		[168]
						40%	3.9 ^a^		
						60%	5.2 ^a^		
						80%	6.2 ^a^		
						100%	7.0 ± 1.3		
	Central	US	Isometric			100%	7.0	Range: 8.0 – 21.0	[152]
	Central	US	Isometric			10Nm		32 0.0 ± 4.0	[151]
	Central	US	Isometric			100%	3.3 ± 0.8		[169]
	Central	US (3D)	Isometric			5%	1.0 ^a^	− 2.4^	[150]
						10%	1.8 ^a^	2.3^	
						25%	2.6 ^a^	10.5^	
						50%	3.7 ^a^	14.5^	
	Central	US	Isometric		Short	15%	1.6 ± 0.4		[157]
						35%	2.5 ± 0.5		
					Moderate	15%	1.1 ± 0.3		
						35%	1.7 ± 0.4		
					Long	15%	0.7 ± 0.2		
						35%	1.1 ± 0.3		
	Central	US	Isometric			100%	1.6 ^c^		[170]
	Central	US	Concentric	20⁰∙s^−1^	120⁰ PF—80⁰ DF	100%	− 1.3 ^c^		[170]
	Central	US	Eccentric	20⁰∙s^−1^	80⁰ DF to 120⁰ PF	100%	0.7 ^c^		[170]
	Anterior	US	Isometric			100%	3.0 ± 0.5		[169]
Vastus lateralis	Deep	US	Isometric			10%	0.5 ^a^		[171]
						20%	1.4 ^a^		
						30%	2.2 ^a^		
						40%	2.9 ^a^		
						50%	3.7 ^a^		
						60%	4.4 ^a^		
						70%	5.1 ^a^		
						80%	6.0 ^a^		
						90%	7.0 ^a^		
						100%	7.4 ^a^		
	Deep	US	Isometric			25%	2.7		[144]
						50%	3.8 ^a^		
						75%	4.6 ^a^		
Rectus Femoris	Posterior^d^	MRI	Concentric	40 cycles/min	150⁰ KE (angular displacement 28 ± 9⁰)	Mean peak load: 5.2 kg/F (at peak stretch)	− 1.6%		[172]
Semitendinosus	Proximal	US	Isometric		Long	10%	6.4 ^a^		[173]
						20%	7.0 ^a^		
						30%	8.0 ^a^		
						40%	8.9 ^a^		
						50%	10.6 ^a^		
						60%	10.8 ^a^		
						70%	10.9 ^a^		
						80%	11.1 ^a^		
						90%	11.8 ^a^		
						100%	11.8 ^a^		
					Moderate	10%	0.0 ^a^		
						20%	0.0 ^a^		
						30%	− 0.2 ^a^		
						40%	0.4 ^a^		
						50%	1.3 ^a^		
						60%	0.9 ^a^		
						70%	1.5 ^a^		
						80%	1.2 ^a^		
						90%	3.0 ^a^		
						100%	5.8 ^a^		
					Short	10%	− 4.2 ^a^		
						20%	− 3.7 ^a^		
						30%	− 2.5 ^a^		
						40%	− 2.3 ^a^		
						50%	− 2.1 ^a^		
						60%	− 2.1 ^a^		
						70%	− 1.3 ^a^		
						80%	− 0.1 ^a^		
						90%	1.3 ^a^		
						100%	2.0 ^a^		
	Distal	US	Isometric		Long	10%	5.6 ^a^		[173]
						20%	4.8 ^a^		
						30%	4.7 ^a^		
						40%	4.9 ^a^		
						50%	4.0 ^a^		
						60%	3.0 ^a^		
						70%	3.1 ^a^		
						80%	2.8 ^a^		
						90%	2.2 ^a^		
						100%	3.7 ^a^		
					Moderate	10%	− 0.1 ^a^		
						20%	− 0.9 ^a^		
						30%	− 1.0 ^a^		
						40%	− 1.3 ^a^		
						50%	− 2.4 ^a^		
						60%	− 4.4 ^a^		
						70%	− 5.6 ^a^		
						80%	− 5.7 ^a^		
						90%	− 4.5 ^a^		
						100%	− 3.8 ^a^		
					Short	10%	− 3.8 ^a^		
						20%	− 4.4 ^a^		
						30%	− 4.8 ^a^		
						40%	− 5.3 ^a^		
						50%	− 6.7 ^a^		
						60%	− 7.1 ^a^		
						70%	− 8.2 ^a^		
						80%	− 8.4 ^a^		
						90%	− 8.0 ^a^		
						100%	− 8.8 ^a^		
	Central aponeurotic inscription (Long arm)	US	Isometric		Long	10%	− 20.6 ^a^		[173]
						20%	− 19.7 ^a^		
						30%	− 14.1 ^a^		
						40%	− 8.2 ^a^		
						50%	− 2.9 ^a^		
						60%	− 0.3 ^a^		
						70%	0.2 ^a^		
						80%	4.6 ^a^		
						90%	7.2 ^a^		
						100%	8.5 ^a^		
					Moderate	10%	− 0.1 ^a^		
						20%	2.2 ^a^		
						30%	2.7 ^a^		
						40%	10.6 ^a^		
						50%	15.3 ^a^		
						60%	19.8 ^a^		
						70%	26.3 ^a^		
						80%	32.6 ^a^		
						90%	35.9 ^a^		
						100%	38.5 ^a^		
					Short	10%	13.9 ^a^		
						20%	17.6 ^a^		
						30%	21.4 ^a^		
						40%	23.5 ^a^		
						50%	28.2 ^a^		
						60%	28.4 ^a^		
						70%	32.1 ^a^		
						80%	40.3 ^a^		
						90%	48.0 ^a^		
						100%	50.1 ^a^		
	Central aponeurotic inscription (Short arm)	US	Isometric		Long	10%	− 14.5 ^a^		[173]
						20%	− 13.2 ^a^		
						30%	− 12.2 ^a^		
						40%	− 11.2 ^a^		
						50%	− 8.7 ^a^		
						60%	− 4.8 ^a^		
						70%	− 3.7 ^a^		
						80%	0.1 ^a^		
						90%	2.5 ^a^		
						100%	4.0 ^a^		
					Moderate	10%	− 0.2 ^a^		
						20%	− 0.7 ^a^		
						30%	0.0 ^a^		
						40%	1.6 ^a^		
						50%	3.9 ^a^		
						60%	7.1 ^a^		
						70%	6.7 ^a^		
						80%	8.6 ^a^		
						90%	11.0 ^a^		
						100%	12.1 ^a^		
					Short	10%	16.0 ^a^		
						20%	14.9 ^a^		
						30%	16.3^a^		
						40%	20.2^a^		
						50%	22.7^a^		
						60%	23.3^a^		
						70%	26.8^a^		
						80%	25.6^a^		
						90%	27.1^a^		
						100%	28.4^a^		
Biceps Femoris Long Head	Proximal aponeurosis	US	Isometric			10%	Proximal: 0.9^a^ Central: 1.3^a^ Distal: 1.2^a^		[174]
						20%	Proximal: 1.6^a^ Central: 3.8^a^ Distal: 2.0^a^		
						30%	Proximal: 2.8^a^ Central: 5.3^a^ Distal: 3.3^a^		
						40%	Proximal: 6.8^a^ Central: 3.6^a^ Distal: 3.9^a^		
						50%	Proximal: 4.9^a^ Central: 3.8^a^ Distal: 7.8^a^		
						60%	Proximal: 5.6^a^ Central: 3.8^a^ Distal: 8.1^a^		
						70%	Proximal: 6.0^a^ Central: 4.1^a^ Distal: 8.5^a^		

Biaxial aponeurosis strain is defined as the deformation of the aponeurosis relative to its resting position both in the longitudinal and transverse directions.

Values expressed as mean ± SD unless stated otherwise; Positive and negative strain indicate lengthening and shortening, respectively. ^a^approximated from digitisation of graphical data; ^b^ Anterior aponeurosis in the region it coalesces with the posterior aponeurosis of the soleus; ^c^ Estimated on average aponeurosis length of 17 cm [167, 168]; ^d^ rectus femoris aponeurosis in the distal region it coalesces with the vastus intermedius aponeurosis; 3D = 3 dimensional; US = ultrasound; MRI = magnetic resonance imaging; MTU = Muscle Tendon Unit; MVIC = maximum voluntary isometric contraction; Kg/F = Kilograms/Force.

**Table 4:** Biaxial stiffness values of human lower-limb aponeuroses

Muscle group	Aponeurosis	Measurement technique	Contraction mode	MTU length	Contraction intensity (MVIC)	Longitudinal stiffness	Transverse stiffness	Reference
Medial Gastrocnemius	Anterior	US/Load Cell^a^	Isometric		100%	Achilles’ tendon repair: Median = 139.59 N/mm (95% CI 115.02—171.21) Control: Median = 414.72 N/mm (95% CI 306.50—462.43)		[175]
	Anterior	US/Load Cell^a^	Isometric		100%	Achilles’ tendon repair: median 374 N/mm (range 88.3–723.7) Control: median 562.1 N/mm (range 166.1–1485.2)		[176]
	Anterior	Uniaxial tensile test (cadaver)^b^	Passive			Proximal: 107.1 ± 33.3 N/mm Central: 125.4 ± 40.1 N/mm Distal: 143.6 ± 45.2 N/mm	Proximal: 0.9 ± 0.9 N/mm Central: 0.6 ± 0.5 N/mm Distal: 0.8 ± 1.1 N/mm	[118]
	Posterior	Uniaxial tensile test (cadaver)^b^	Passive			Proximal: 114.2 ± 36.7 N/mm Central: 114.3 ± 37.5 N/mm Distal: 83.3 ± 34.8 N/mm	Proximal: 0.7 ± 1.4 N/mm Central: 0.4 ± 0.7 N/mm Distal: 0.3 ± 0.4 N/mm	[118]
Lateral gastrocnemius	Anterior	Uniaxial tensile test (cadaver)^b^	Passive			Proximal: 102.0 ± 45.9 N/mm Central: 144.7 ± 41.4 N/mm Distal: 129.6 ± 46.7 N/mm	Proximal: 0.8 ± 0.9 N/mm Central: 1.2 ± 1.3 N/mm Distal: 1.8 ± 1.6 N/mm	[118]
	Posterior	Uniaxial tensile test (cadaver)^b^	Passive			Proximal: 108.2 ± 35.0 N/mm Central: 109.7 ± 36.1 N/mm Distal: 87.5 ± 18.7 N/mm	Proximal: 0.7 ± 0.6 N/mm Central: 0.9 ± 1.0 N/mm Distal: 0.8 ± 0.5 N/mm	[118]
Soleus	Anterior	Uniaxial tensile test (cadaver)^b^	Passive			Medial: 73.9 ± 4.9 N/mm Lateral: 76.6 ± 3.2 N/mm	Medial: 0.9 ± 0.1 N/mn Lateral: 1.0 ± 0.4 N/mm	[118]
	Posterior	Uniaxial tensile test (cadaver)^b^	Passive			Medial: 93.4 ± 12.1 N/mm Lateral: 77.9 ± 12.2 N/mm	Medial: 0.9 ± 0.1 N/mm Lateral: 1.3 ± 0.2 N/mm	[118]
Tibialis anterior	Central	US/IKD	Isometric	Short	15%	30 ± 16 N/mm		[157]
					35%	68 ± 15 N/mm		
				Neutral	15%	47 ± 26 N/mm		
					35%	97 ± 48 N/mm		
				Long	15%	68 ± 28 N/mm		
					35%	134 ± 69 N/mm		

Aponeurosis stiffness describes the change in aponeurosis dimensions (biaxial deformation) in relation to the force applied to the aponeurosis. This property is dependent on the dimensions of the aponeurosis (greater CSA and shorter length (i.e. increased thickness) may lead to greater stiffness [117, 118]).

Values expressed as mean ± SD unless stated otherwise; Positive and negative strain indicate lengthening and shortening, respectively. ^a^ In vivo measurement methods, ^b^ Ex vivo measurement methods. 3D, 3 dimensional; IKD, isokinetic dynamometer; MRI, magnetic resonance imaging; MTU, Muscle Tendon Unit; MVIC, maximum voluntary isometric contraction; US, ultrasound.

**Table 5:** Biaxial modulus of human lower-limb aponeuroses

Muscle	Aponeurosis	Measurement technique	Contraction mode	Longitudinal modulus (MPa)	Transverse modulus (MPa)	References
Medial Gastrocnemius	Anterior	Uniaxial tensile test (cadaver)	Passive	Proximal: 198.2 ± 118.3 Central: 196.1 ± 89.2 Distal: 207.5 ± 103.2	Proximal: 1.1 ± 1.2 Central: 0.8 ± 0.9 Distal: 0.5 ± 0.5	[118]
	Posterior	Uniaxial tensile test (cadaver)	Passive	Proximal: 207.1 ± 118.7 Central:210.4 ± 96 Distal: 182.8 ± 106.6	Proximal: 0.6 ± 0.9 Central: 0.4 ± 0.6 Distal: 0.6 ± 0.6	
Lateral Gastrocnemius	Anterior	Uniaxial tensile test (cadaver)	Passive	Proximal: 283.9 ± 168.9 Central:323.3 ± 151 Distal: 304.6 ± 157.1	Proximal: 1.1 ± 1.1 Central:1.4 ± 1.4 Distal: 1.9 ± 1.9	
	Posterior	Uniaxial tensile test (cadaver)	Passive	Proximal: 289.1 ± 214.5 Central:280 ± 104.4 Distal: 177.6 ± 120.0	Proximal: 0.5 ± 0.5 Central:1.0 ± 1.2 Distal: 1.3 ± 0.7	
Medial Soleus	Anterior	Uniaxial tensile test (cadaver)	Passive	Proximal: 211.6 ± 166.1 Central:199.1 ± 113.1 Distal: 191.9 ± 66.5	Proximal: 0.7 ± 0.9 Central:0.8 ± 0.9 Distal: 0.8 ± 0.5	
	Posterior	Uniaxial tensile test (cadaver)	Passive	Proximal: 264.3 ± 155.5 Central:261.7 ± 196.9 Distal: 262.0 ± 127.7	Proximal: 1.2 ± 1.0 Central:1.4 ± 1.6 Distal: 0.9 ± 0.9	
Lateral Soleus	Anterior	Uniaxial tensile test (cadaver)	Passive	Proximal: 164.1 ± 124.6 Central:197.9 ± 181.2 Distal: 173.8 ± 75.6	Proximal: 0.8 ± 0.9 Central:0.7 ± 0.5 Distal: 1.3 ± 1.4	
	Posterior	Uniaxial tensile test (cadaver)	Passive	Proximal: 185.7 ± 132.2 Central:225.4 ± 168.8 Distal: 256.2 ± 159.2	Proximal: 2.3 ± 2.2 Central:2.0 ± 1.9 Distal: 1.9 ± 1.9	

Elastic modulus of the aponeurosis describes the relationship between aponeurosis stress and strain, representing the intrinsic properties of the aponeurosis material, independent of CSA.

Values expressed as mean ± SD unless stated otherwise; CSA, Cross sectional area; MPa, Megapascal*.*

Aponeurosis mechanical behaviour during muscle contraction is a function of the muscle length change and activation level over time—muscle force is dependent on these two parameters that translates to the load on the aponeurosis that causes deformation. Several factors influence this complex deformation: such as aponeurosis micro and macro-scale morphology, contraction type, contraction intensity, contraction mode, MTU length, and neural drive. Collagen structure will determine tissue-level mechanical properties of the aponeuroses. Both macro-scale morphology (geometry) and contractile conditions will not change the underlying material properties but will impact the mechanical behaviour.

### Aponeurosis Structure

When discussing aponeurosis strain magnitude and comparing regions of an aponeurosis or between the aponeuroses of different MTUs, it is important to discuss the context of the muscle action (e.g., isometrically contracting, passively lengthening). Without the context of the in vivo conditions of a particular MTU, it is difficult to discern how the unique morphology of the aponeurosis vs. a possible differential muscle action may lead to the observed behaviour of the muscle fibres and aponeuroses. Despite this complexity, the variable structure of the aponeuroses of MTUs in the lower limb likely contributes to the non-uniform mechanical behaviour displayed within the same aponeurosis [91, 117, 177, 178]. For instance, smaller magnitudes of strain are demonstrated at the tendon-aponeurosis junction, where the aponeurosis is thicker. This is the case for the posterior aponeurosis of the soleus in that a 2.2% increase in length was recorded in the thinner mid region compared to a 2.5% decrease at the thicker distal region [166] during sub-maximal isometric conditions. Similar regional differences were also reported for the medial gastrocnemius, and BFlh during submaximal isometric contractions (10–40% MVIC) [159, 174] as well as during passive lengthening in cadaveric specimens of the triceps surae [118]. Contrastingly, the BFlh proximal aponeurosis strain pattern was reversed as contraction intensity increased (40–70% MVIC) in that both the thicker proximal and distal regions appeared to strain more than the middle region.

The aponeurosis also appears to undergo a non-uniform strain in response to mechanical load highlighted by the fact that regions of the medial gastrocnemius aponeuroses in fact shortened under load [159]. One possible reason for this non-uniform strain pattern is the complex geometry of the interdigitating aponeurotic morphology between muscles (e.g., the central (intramuscular) soleus aponeurosis – the anterior projection of the posterior aponeurosis [179]) [91, 134, 180]. Another theory suggests the non-uniform strain behaviour may be due to the tight mechanical interaction between the aponeurosis and muscle fibres [47, 153, 159, 166, 179]. Upon shortening contraction of muscle tissue during active force production, the aponeurosis undergoes large transverse stretch and may result in subsequent longitudinal shortening of the aponeuroses [47, 153, 159, 166, 179] and alterations in the pennation of muscle fascicles at these sites of aponeurosis shortening [178].

There are fewer data regarding transverse strain of the lower-limb aponeuroses compared to longitudinal strain. In one study of aponeurotic tissue of the medial gastrocnemius at the point where the anterior medial gastrocnemius and posterior soleus aponeuroses coalesce, transverse strain ranged from 8.5 to 13.9% (60% MVIC), which was overall higher than longitudinal strain values in this muscle group [147]. There appeared to be no significant difference in transverse strain between thinner proximal and thicker distal portions of the anterior aponeurosis of medial gastrocnemius [147].

Regional variation of longitudinal mechanical stiffness in the gastrocnemius aponeurosis was demonstrated during passive lengthening, but notably at the proximal, middle and distal sites the elastic modulus showed no differences in any regions [118]. Shan et al.[118] speculated that the regional differences in aponeurosis mechanical stiffness may be due to a difference in thickness rather than a difference in the material property. Whilst this is an accurate description of mechanical stiffness it likely does not best represent the stiffness of the aponeurosis under in vivo conditions due to the method of separating the aponeurosis from the other active and passive elements of the MTU.Further work is also required to fully understand if the ultrastructure of aponeurotic tissue is different between regions, muscles and/or individuals.

### Contractile Conditions

#### Passive and Active Loading

When placed under active loading conditions, human aponeuroses tend to demonstrate greater magnitudes of strain compared to passive loading conditions. For example, the anterior aponeurosis of the medial gastrocnemius demonstrates longitudinal strains of − 3% to 9% during isometric loading conditions (20–100% MVIC) [158–161], and − 2 to 4% under passive lengthening (5°∙s^−1^) [162, 163]. The variable contraction intensity between studies likely impacts these findings with strain during lower contraction intensity (< 30% MVIC) [158–161] being more comparable to the strain during passive lengthening [162, 163]. This may suggest the strain behaviour of the aponeurosis is variable during high and low intensity muscle contraction. There are fewer homogenous data [158–161, 163] regarding the influence of contraction type on human aponeurosis stiffness and modulus, making it difficult to synthesise the evidence..

#### Contraction Intensity

Contraction intensity influences aponeurotic longitudinal strain, with higher strain values at greater contraction intensities during isometric contractions. At 90–100% MVIC, longitudinal strain of the anterior aponeurosis of the medial gastrocnemius typically ranged from 4 to 7% [158, 160, 164], compared to 1–4% at 10–30% MVIC [147, 164]. This pattern of increasing longitudinal strain with increasing contraction intensities is also reflected in the central aponeurosis of tibialis anterior [150, 153, 168, 170], deep aponeurosis of vastus lateralis [144] and the proximal aponeurosis of BFlh [174] (Table 3). The longitudinal strain of the proximal aponeurosis of semitendinosus also increases with the contraction intensity while that of the distal aponeurosis decreases with increasing contraction intensity [173] (Table 3). Similar to the influence of contraction intensity on aponeurotic longitudinal strain, the transverse strain of the central aponeurosis of tibialis anterior also increased with contraction intensity from − 2% at 5% MVIC to 15% at 50% MVIC [150], as did the anterior aponeurosis of medial gastrocnemius at 30% and 60% MVIC (proximal: 8–9%, distal: 11–14%) [147].

Distribution of strain within the aponeurosis is likely to be influenced by neural drive to the muscle. Aponeuroses strain nonuniformly along their length during isometric contractions [159, 166], which may arise because of regional activation differences due to the spatial distribution of active motor units [159, 166, 181]. Differential muscle activation between and within muscle compartments may induce considerable heterogeneity throughout the length and width of the muscle and subsequently influence aponeurosis deformation [166, 182–184]. In addition, regional muscle activation can also be influenced by muscle fatigue and exercise-induced muscle damage that might also alter the distribution of strain within the aponeurosis [185, 186].

Increasing contraction intensity also appears to lead to an increase in the apparent longitudinal stiffness in the central aponeurosis of the tibialis anterior [153]. It has been suggested that the increases in aponeurosis width with increasing contraction intensity likely influence the longitudinal stiffness of the structure within the MTU [94, 150, 157, 174]. This may be because at higher muscle forces there is greater muscle fibre bulging and intramuscular pressure leading to increases in transverse stretch and longitudinal shortening of the aponeurosis [150, 153]. It is also important to note that for nonlinear tissues like the aponeurosis, apparent stiffness will increase with strain level. So, the fact that the perceived stiffness is greater at higher intensities may simply be due to the nonlinear nature of the material.

#### Contraction Mode

Only two studies have investigated how concentric [170, 172] or eccentric [170] contraction modes influence aponeurosis behaviour. Most studies investigating aponeurosis behaviour have generally investigated (via b-mode ultrasound) isometric contractions that demonstrated a linear response to increasing muscle force [147, 150–152, 166, 174]. Length changes of the tibialis anterior central aponeurosis have been shown to behave differently between contraction modes [170]. The aponeurosis shortened during concentric contractions about twice as much as the lengthening of the aponeurosis during eccentric contractions (− 2.2 and 1.0 mm, respectively), when a dorsiflexion torque of approximately 27 Nm was applied [170]. Similarly, within the quadriceps femoris, the aponeurosis (where the rectus femoris and vastus intermedius coalesce) shortened 1.6% during cyclic concentric contractions [172].

Possible explanations for the greater shortening during concentric contractions might be due to the aponeurosis being interconnected with muscle fibers [46, 60], the isovolumetric nature of muscle tissue during shortening contractions [146] and the ratio of transverse to longitudinal strain under increasing active force production [94, 151]. Alternatively, differences in mechanical behaviour between short and long MTU lengths during isometric contractions may influence transverse strain magnitude and the subsequent apparent longitudinal stiffness may provide some insight into the difference in length change between concentric and eccentric contractions [151, 152]. However, these changes in starting MTU length during isometric contractions can only partially explain the disparity between contraction modes. Further work is necessary to identify how active shortening and lengthening may affect the mechanical behaviour of the aponeurosis. This work must also consider changes in muscle architecture, contraction velocity and intensity during eccentric and concentric contractions.

#### Muscle–Tendon Unit Length

The length of the MTU relative to its slack length appears to influence the aponeurotic strain response. Under passive loading conditions, the anterior aponeurosis of the medial gastrocnemius demonstrated significantly higher longitudinal strain values in dorsiflexion (3%) compared to plantarflexion (− 2%) [162, 163]. Under isometric loading (10–100% MVIC), the proximal and distal aponeuroses of semitendinosus tended to increase in strain at longer MTU lengths [173]. The inverse was true for the central aponeurotic inscription of semitendinosus which appeared to strain more at shorter MTU lengths compared to longer MTU lengths [173]. This was consistent with the central aponeurosis of the tibialis anterior (15–35% MVIC) that demonstrated greater longitudinal strain in dorsiflexion (3%) compared to plantarflexion (1%) [153]. Other work observing the central aponeurosis of the tibialis anterior found greater transverse strain at short compared to long MTU lengths [151, 152]. These findings may suggest a difference in strain behaviour between the intra-and-extra-muscular aponeuroses during isometric contractions.

A pattern of increasing apparent longitudinal stiffness in the central aponeurosis of tibialis anterior [153] with increasing MTU length during active contraction has also been reported. For the tibialis anterior, it was postulated that during shortening contractions, increases in intramuscular pressure and fiber bulging acted to stretch the aponeurosis in the transverse direction [94, 150, 157]. As the MTU shifted from a short to a longer length, the aponeurosis stretched in the transverse direction relatively more than the longitudinal direction as muscle force increased [157]. As a result, such increases in aponeurosis width at increasing length and contraction intensity led to longitudinal shortening of the aponeurosis and therefore contributed to the increased longitudinal stiffness.

### Future Directions

Several factors were identified in this review that influence the mechanical properties of the human aponeuroses, including contraction type, loading intensity, MTU length and muscle activation. Additionally, it appears aponeurosis mechanical behaviour is muscle specific and practitioners should be careful to not conflate the behaviour from one myo-aponeurotic complex with others. To fully understand the behaviour of the human aponeuroses, consistency of measurement methods to best quantify the mechanical properties of individual aponeuroses is required. Due to the three-dimensional deformation of muscle and aponeurotic tissue, imaging methods across multiple planes such as three-dimensional ultrasound [150, 157], MRI [159, 179] and image-based computational modelling [117, 131, 187] appear to be best practice. These techniques allow estimation of the internal tissue response across a wide range of loading intensities, contraction modes and functional tasks and are not bound by in-series or in-parallel designations. These techniques could also include analysis of movement tasks that are commonly associated with injury [188] as well as exercises that inform injury prevention [189] and rehabilitation interventions [10].

## Adaptation of Aponeuroses to Mechanical Stimuli

Despite extensive research on muscle and tendon adaptation, the adaptive response for the aponeurosis to mechanical stimuli remains under researched. Skeletal muscle and tendon tissue have been shown in great detail to undergo regular cellular turnover in response to changes in mechanical stimuli [190–195]. These physiological changes are largely determined by the morphological, mechanical and material properties along with the current load tolerance of the tissue [15, 190, 196–198]. It is well accepted that mechano-transduction mechanisms induced by mechanical load influence the homeostasis of connective tissue to regulate the adaptation process [190, 199–202]. Furthermore, the modulation of the mechanical stimuli (e.g., overload or underload, high strain or low strain tasks) can influence the effectiveness of the tissue adaptative response to exercise [190, 203]. The following sections provide a summary of aponeurosis adaptation in response to a variety of mechanical stimuli. Studies of the free tendon and aponeurosis in combination were not included, as the mechanical behaviour of the aponeurosis in isolation is likely to differ. While it is likely that the mechano-transduction mechanisms in aponeurosis and free tendon are similar, the stimuli driving the adaptive response will vary substantially given the difference in their mechanical behaviour. We acknowledge that hormonal, neural, nutritional, and metabolic factors may also influence the adaptation of aponeurotic tissue; however, these factors were beyond the scope of this review.

### Adaptation of Morphological Properties

Morphological adaptation of the deep aponeurosis of the vastus lateralis muscle has been examined in two studies [136, 137]. Both studies measured morphological variables pre and post a 12-week training program of unilateral knee extension exercises and compared results to an untrained control group (which did not demonstrate a significant change).

One study demonstrated a significant increase in aponeurotic width (2%) [136] following loaded knee extension through ~ 20–100° of knee flexion (five sets of eight repetitions at a load of 80% of one-repetition maximum) three times a week across a 12-week period (for a total of 1,440 repetitions) [136]. Meanwhile the other study demonstrated a significant increase in aponeurotic cross sectional area (7%) [137] following sustained isometric knee extension contractions (75% maximal voluntary torque (MVT) holds for 3 s). The intervention involved 40 unilateral isometric knee extension contractions (four sets of ten) three times a week over a 12-week period for both training groups (1,440 total contractions). An explosive isometric knee extension (~ 80% MVT) as well as a non-training control group showed no significant changes in aponeurosis morphology [137].

Aponeurotic morphological adaptation in response to prolonged resistance training appears to correlate with muscular morphological adaptation, and probably serves to provide a greater myo-aponeurotic interface area for muscle fibre attachment. For example, significant increases in the aponeurotic width (2%) after loaded knee extension exercises was accompanied by a significant increase in muscle cross sectional area (11%) [136]. Similarly, an increase in aponeurotic area (7%), was also accompanied by an increase in vastus lateralis volume after sustained isometrics (8%) but not after explosive isometrics (3%) [137]. However, it is unclear if this association holds true for other muscles (e.g., BFlh) due to the variable myo-aponeurotic morphology between different muscles.

These findings are supported by cross-sectional studies, although it is not possible to infer causality from cross-sectional study designs. The area of the deep vastus lateralis aponeurosis in Olympic-level weightlifters for instance was 32% greater than that of the recreationally active students (no differences in age, standing height, and fat percentage between the two groups), with a strong correlation between quadriceps muscle volume and aponeurosis area (r = 0.85) [138]. Similarly, long-term resistance trained individuals had a 17% larger VL aponeurosis area than untrained controls [204]. Interestingly, there was no significant difference in tendon cross sectional area between the groups [204].

Unloading protocols had a similar effect on morphology as mechanical overload. After 4 weeks of dominant lower leg unloading using a sling, the distal aspect of the posterior soleus aponeurosis demonstrated a significant increase in cross sectional area at several regions [205]. The overall aponeurosis volume also tended to increase (6%) but did not reach significance (*p* = 0.06) [205]. The authors suggested increases in water content within the ECM and fluid shift during limb suspension may potentially explain this hypertrophic effect [205].

### Adaptation of Mechanical and Material Properties

Mechanical overload has been shown to induce an adaptative response in the mechanical and material properties (e.g., stress, strain, stiffness and elastic modulus) of the composite tendon-aponeurosis [15, 137, 140, 206–208]. However there have been no studies that have examined the effects of mechanical overload on the aponeurosis in isolation. There has been research into the effects on unloading of the posterior soleus aponeurosis in isolation. One study demonstrated that unloading of this aponeurosis over 4 weeks using lower leg suspension resulted in a significant reduction in mechanical stress by 44.5%[205].

Another study demonstrated that the posterior soleus aponeurosis changed from a non-uniform strain pattern to a more uniform distribution after the same unloading intervention [179]. This uniform strain pattern was due to the mid-region of the posterior soleus aponeurosis changing from positive strain (lengthening) to negative strain (shortening) during sub-maximal contraction (20% MVIC). The authors speculated this adaptation may be a result of regional changes in myo-aponeurotic stiffness, muscle architecture or motor unit recruitment strategies [179].

Significant reductions (29%) of elastic modulus within the posterior soleus aponeurosis were associated with 4 weeks of limb suspension [205]. These findings may be due to changes to the intrinsic collagen structure and/or composition of the aponeurosis [84, 101, 199, 209] and/or potentially due to an increase in water content within the ECM [19, 205].

### Future Directions

Whilst there is some research on the effect of mechanical overload on human aponeurosis morphology, the effects on aponeurotic mechanical properties remain unclear. Long term (> 12 weeks) mechanical overload (e.g., via high intensity, high-strain resistance training) appears to result in increases in aponeurotic width and cross-sectional area, albeit according to only two intervention studies [136, 137]. Further, large reductions in elastic modulus were associated with unloading (zero-strain) protocols. Additionally, the aponeurosis non-uniform strain pattern is potentially reversed by unloading that induces negative changes in mechanical stress and modulus. However, caution should be exercised when interpreting these changes, as they have only been investigated in single studies thus far [179, 205]. Whilst this work may suggest the aponeurosis has some capacity for morphological adaptation, the muscle specific nature of aponeurotic structure–function relationships makes it challenging to infer the adaptive response to all aponeuroses. For example, morphological adaptation of the deep aponeurosis of the vastus lateralis likely does not reflect the adaptive properties of other lower limb aponeuroses due to differences in the myo-aponeurotic architecture. Future research should aim to investigate the effects of mechanical overload and/or detraining on specific muscles and their associated aponeurotic structure/s. Additionally, future studies should aim to observe the adaptive response of the mechanical and material properties of the aponeurosis separate from the free tendon. For example, interventional studies should address a range of contraction modes such as eccentric training, a recommended intervention for hamstring injury prevention [10, 189, 210, 211], as well as a variety of loading intensities, strain magnitudes and contraction velocities that are commonly implemented in injury prevention and rehabilitation programs.

## Considerations for Myo-Aponeurotic Injury Risk

The aponeurotic morphological, mechanical, and material properties described above do not currently represent an independent risk factor for injury. Instead, aponeurotic injury risk is more complex, requiring consideration of the interaction between muscle-aponeurosis-tendon geometry, muscle architecture, and connective tissue properties. Image-based muscle modelling approaches can provide further insight to better understand how these factors may influence strain behaviour and potentially provide a framework for future large scale prospective studies assessing risk factors for myo-aponeurotic injury.

Retrospective MRI data suggest that a smaller aponeurosis CSA may increase hamstring strain injury risk due to a greater concentration of stress and strain at the myo-aponeurotic interface [127]. The authors found that the aponeurosis-to-muscle cross sectional area ratio exhibited six-fold variability across individuals [127]. Given this inherent variability, inter-individual differences in aponeurosis-to-muscle CSA ratio, the proximal aponeurosis was proposed as a factor that could modulate risk of injury. This is consistent with cadaveric and other MRI findings that have also demonstrated marked variation among the proximal hamstring myo-aponeurotic morphology [125].

Additionally, computational modelling studies found that a narrower proximal BFlh aponeurosis was associated with greater muscle fibre strain during active lengthening conditions and high-speed running [115, 116]. Fiorentino et al. [115] also demonstrated that the ratio of widths between the BFlh proximal aponeurosis and muscle tissue also influences muscle fibre strain magnitude. As such, larger peak local strains were observed with a narrow aponeurosis whilst the inverse is true of muscle width during high-speed running. These findings are significant because active lengthening and high-speed running are common mechanisms of myo-aponeurotic injuries [188, 212–214], and help to understand how the influence of aponeurosis morphology may potentially risk-stratify athletes.

A recent retrospective study also found variation in the morphology of the proximal BFlh aponeurosis across all athletes according to MRI [128]. Whilst the investigators found no significant difference in the width, length or CSA of the aponeurosis between injured and uninjured legs, the injured group demonstrated a larger aponeurotic volume, compared to the uninjured group [128]. The increased proximal aponeurosis volume may be due to an increased thickness of the aponeurosis in response to healing, as has been observed previously [18, 82, 128]. It is conceivable that a change in thickness may also influence the stiffness properties of the aponeurotic structure [117]. That is, a thicker proximal aponeurosis [117, 128, 215] may result in greater muscle fibre strain during contraction [117], that may have some bearing on re-injury risk. Modelling studies support this as they have shown that the morphology (or geometry) of an aponeurosis directly influences the strain on the attaching muscle fibres [116, 117, 216, 217]. Rehorn and Blemker [117] studied the effects of varying width and thickness of the BFlh aponeurosis and found that a narrower and thicker aponeurosis leads to greater along-fibre strain during lengthening contractions. Further, aponeurosis width was found to influence strain distribution –a narrower proximal BFlh aponeurosis causes greater strain at the proximal myo-aponeurotic junction [115–117]. This finding may have important implications for our understanding of injury risk because greater muscle fibre strain at the myo-aponeurotic junction may increase the likelihood of injury at this site [100, 112, 117, 218].

## Conclusions

The aponeuroses of lower-limb muscles are intricate and demonstrate marked variation within and between muscles. This makes comparison between MTUs challenging and therefore future research should aim to determine the mechanical behaviour and adaptation specific to individual aponeuroses. Notable variation also appears between regions and individuals which potentially has implications for individual specific mechanical behaviour and propensity for injury. Several factors appear to influence the deformation of lower-limb aponeuroses, including macro-scale morphology, collagen structure, contraction mode, MTU length, and muscle activation. Due to the muscle specific behaviour of the aponeurosis, future research could focus on the individual aponeurotic tissue response to load, particularly in those prone to injury. Image-based modelling approaches appear best placed to begin to answer some of these questions considering the aponeurosis is not well defined by explicit series or parallel designations. This review also identified a notable lack of data regarding the adaptive properties of key lower-limb aponeuroses. Future studies should focus on the potential capacity for morphological, mechanical, and material changes following mechanical overload or underload for specific MTUs and their related aponeurotic structure. Finally, myo-aponeurotic injuries of the lower limb are commonly involved during high strain-type mechanisms. Due to the often-prolonged rehabilitation and return-to-sport timeframes, future research should focus on strategies to prevent primary and recurrent injuries that involve the aponeurosis.

## Data Availability

Not applicable. The data that support the findings of this paper are available from the cited research articles.

## Abbreviations

3D: 3 Dimensional
BFlh: Biceps femoris long head
BFsh: Biceps femoris short head
BAMIC: British Athletics Muscle Injury Classification
CSA: Cross sectional area.
ECM: Extra-cellular matrix
Kg/F: Kilograms/Force
IKD: Isokinetic dynamometer
MRI: Magnetic resonance imaging
MVT: Maximal voluntary torque
MVIC: Maximum voluntary isometric contraction
MPa: Megapascal
MTJ: Muscle-tendon junction
MTU: Muscle-tendon unit
SM: Semimembranosus
ST: Semitendinosus
US: Ultrasound
VM: Vastus medialis
VI: Vastus intermedius
